# The extracellular domain of site-2-metalloprotease RseP is important for sensitivity to bacteriocin EntK1

**DOI:** 10.1016/j.jbc.2022.102593

**Published:** 2022-10-14

**Authors:** Sofie S. Kristensen, Thomas F. Oftedal, Åsmund K. Røhr, Vincent G.H. Eijsink, Geir Mathiesen, Dzung B. Diep

**Affiliations:** Faculty of Chemistry, Biotechnology, and Food Science, Norwegian University of Life Sciences (NMBU), Ås, Norway

**Keywords:** bacteriocin, enterococcus, membrane protein, metalloprotease, PDZ domain, site-directed mutagenesis, regulated intramembrane proteolysis, drug design, *Ec*RseP, *Escherichia coli* RseP, *Efm*RseP, *Enterococcus faecium* RseP, *Efs*RseP, *Enterococcus faecalis* RseP, EntK1, Enterocin K1, *Lp*RseP, *Lactiplantibacillus plantarum* RseP, MFI, median fluorescence intensity, MIC50, minimal inhibitory concentration, MRE β-loop, membrane-reentrant β-hairpin–like loop, RIP, regulated intramembrane proteolysis, S2P, site-2-metalloprotease, TMS, transmembrane segment

## Abstract

Enterocin K1 (EntK1), a bacteriocin that is highly potent against vancomycin-resistant enterococci, depends on binding to an intramembrane protease of the site-2 protease family, RseP, for its antimicrobial activity. RseP is highly conserved in both EntK1-sensitive and EntK1-insensitive bacteria, and the molecular mechanisms underlying the interaction between RseP and EntK1 and bacteriocin sensitivity are unknown. Here, we describe a mutational study of RseP from EntK1-sensitive *Enterococcus faecium* to identify regions of RseP involved in bacteriocin binding and activity. Mutational effects were assessed by studying EntK1 sensitivity and binding with strains of naturally EntK1-insensitive *Lactiplantibacillus plantarum*–expressing various RseP variants. We determined that site-directed mutations in conserved sequence motifs related to catalysis and substrate binding, and even deletion of two such motifs known to be involved in substrate binding, did not abolish bacteriocin sensitivity, with one exception. A mutation of a highly conserved asparagine, Asn359, in the extended so-called LDG motif abolished both binding of and killing by EntK1. By constructing various hybrids of the RseP proteins from sensitive *E. faecium* and insensitive *L. plantarum*, we showed that the extracellular PDZ domain is the key determinant of EntK1 sensitivity. Taken together, these data may provide valuable insight for guided construction of novel bacteriocins and may contribute to establishing RseP as an antibacterial target.

Site-2-metalloproteases (S2Ps) are a family of intramembrane-cleaving proteases involved in regulated intramembrane proteolysis (RIP) (1, 2). In the RIP cascade, an S2P cleaves its substrate, for example, a membrane-bound anti-sigma factor, within the cell membrane, thereby mediating transmembrane signaling to trigger an adaptive response. S2Ps are conserved in all kingdoms of life and are crucial in several biological processes, including stress response, sporulation, cell polarity, virulence, and nutrient uptake (3, 4, 5, 6, 7, 8, 9). Due to its vital role in both animal and human pathogens, RseP is regarded as an attractive antimicrobial target. In fact, nature itself targets RseP, which is a known target for antimicrobial peptides belonging to the LsbB family of bacteriocins in selected Gram-positive bacteria (10, 11). Little is known about how these bacteriocins recognize and bind RseP and how this interaction eventually leads to killing of target cells. More insight into these issues is crucial for understanding bacteriocin function and for understanding how RseP may be targeted in antimicrobial therapy.

The hallmarks of the S2P family are the conserved catalytic motifs (HExxH and LDG) located on transmembrane segments (TMSs) of the protease (12). The S2P family of proteases is divided into four subgroups based on membrane topology and domain structure (13). Among the four groups, only a few members have been characterized; these include *Escherichia coli* RseP (*Ec*RseP) from group I and the group III members MjS2P and SpolVFB from *Methanocaldococcus jannaschii* and *Bacillus subtilis*, respectively (12, 14, 15). *Ec*RseP is the most extensively studied S2P and was first identified as a key modulator of stress response (16, 17). When *E. coli* cells are exposed to stress, a site-1-protease cleaves the membrane-bound anti-σ^E^ factor RseA. This primary cleavage triggers a secondary cleavage by the S2P *Ec*RseP, which leads to release of RseA into the cytosol (16, 17, 18). RseA is further processed in the cytosol to form the mature σ^E^, which activates genes involved in the stress response (19). It is believed that most S2P signaling pathways follow this same general cascade.

Next to the catalytic motifs, several conserved regions are thought to be involved in substrate interaction and catalysis by *Ec*RseP. These include the membrane-reentrant β-hairpin–like loop (MRE β-loop), the GxG motif, and the PDZ domain (Fig. 1) (20, 21, 22). The PDZ domain has been suggested to work as a size-exclusion filter, preventing interaction with the substrate prior to site-1-protease cleavage (21, 23).

**Figure 1:**
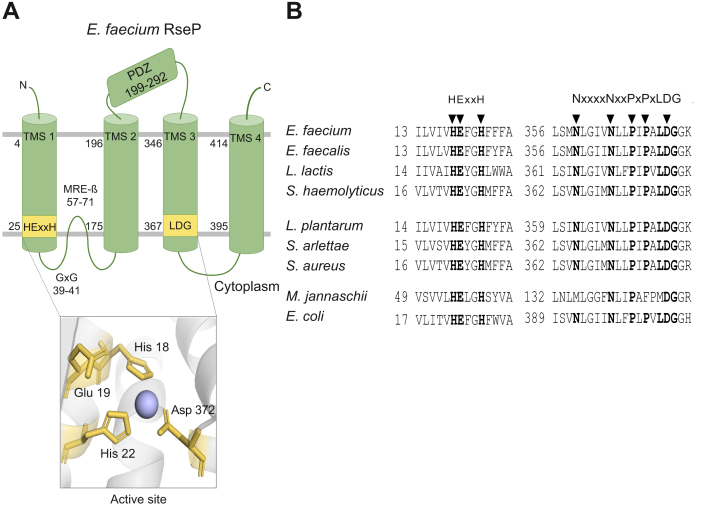
**Schematic representation of the topology of *Efm*RseP and alignment of conserved S2P regions.***A*, schematic representation of the predicted topology of *Enterococcus faecium* RseP with conserved S2P motifs indicated. TMS1-4 indicates the four predicted transmembrane segments. The GxG motif, MRE β-loop, and the predicted PDZ domain are indicated. The *box* below shows the predicted active site, consisting of the conserved HEXXH and LDG motifs. *B*, alignment of the amino acid sequences of active site and the extended LDG motif in RseP from four EntK1-sensitive species (*E. faecium*, *Enterococus faecalis*, *Lactococcus lactis*, and *Staphylococcus haemolyticus*) and three EntK1-insensitive species (*Lactiplantibacillus plantarum*, *Staphylococcus arlettae*, and *Staphylococcus aureus*), as well as Gram-negative *Escherichia coli* and *Methanocaldococcus jannaschii*. *Arrow heads* indicate residues subjected to alanine substitutions. EntK1, Enterocin K1; MRE β-loop, membrane-reentrant β-hairpin–like loop; S2P, site-2-metalloprotease; TMS, transmembrane segment.

In addition, conserved residues near the LDG catalytic motif located in the third transmembrane segment (TMS3) have been implicated in substrate binding and recognition, in particular two asparagines and prolines in the sequence motif NxxxxNxxPxPxLDG (24), here referred to as the extended LDG motif. Despite the identification of these potentially important features, the mechanism of substrate recognition and binding by *Ec*RseP remains somewhat enigmatic.

Bacteriocins are antimicrobial peptides produced by bacteria to inhibit other bacteria in competition for nutrition and ecological niches. They are considered promising alternatives and/or complements to antibiotics, mainly due to their potent activity against multidrug resistant pathogens. We have previously demonstrated that enterocin K1 (EntK1), a leaderless bacteriocin belonging to the LsbB family, is especially potent against *Enterococcus faecium*, including vancomycin-resistant strains (11, 25). Leaderless bacteriocins are synthesized without an N-terminal leader sequence and do not have posttranslational modifications, making this group of bacteriocins ideal for synthetic production. Members of the LsbB family are small (30–44 amino acids), cationic, and amphiphilic, with an N-terminal helical structure and a disordered C-terminal end (11, 26). Interestingly, members of the LsbB family of bacteriocins differ in their inhibition spectrum, with LsbB being active only against *Lactococcus lactis*, while the inhibitory spectrum of EntK1 and enterocin EJ97 (EntEJ97) is broader, including high activity toward *E. faecium* and *Enterococcus faecalis*, respectively (11). It has previously been shown that the antimicrobial activity of bacteriocins of the LsbB family depends on RseP being present in target cells (10, 11).

RseP of *E. faecium* (*Efm*RseP) and *Ec*RseP, both from subgroup 1, shares a 28% sequence identity and has the same predicted membrane topology and conserved domains (Fig. 1). Little is known about the function of RseP in *E. faecium*; however, recent phenotypic analysis of *rseP* mutants suggests a role in stress response (25). For *E. faecalis*, it has been shown that RseP (*Efs*RseP) is a key regulator of the stress response through RIP-mediated activation of the sigma factor SigV. Deletion of either *EfsrseP* or *sigV* increases the susceptibility of *E. faecalis* to multiple stressors, such as lysozyme, heat, ethanol, and acid (27). In addition, *Efs*RseP is involved in sex pheromone maturation and is therefore also referred to as Eep (enhanced expression of pheromone) in this organism (28). Lastly, deletion of *EfsrseP* has been shown to result in severely attenuated virulence in a rabbit endocarditis model and a catheter-associated urinary tract infection model, suggesting an important role for *Efs*RseP in pathogenesis (7, 29).

Despite the evident role of RseP in enterococcal virulence, critical features of enterococcal RseP, such as the substrate recognition mechanism, remain unknown. The known substrates of RseP-like S2P share no apparent sequence homology; however, amphiphilic helices in the substrates have been indicated as necessary for recognition (20, 30). Considering the helical structure of EntK1, it is conceivable that EntK1 interacts with enterococcal RseP in a similar manner as the native substrates. Therefore, to gain more insight into bacteriocin action and possibly the interaction between RseP and its natural substrates, we have studied the EntK1–RseP interaction, focusing on the role of conserved regions of RseP. The impact of mutations in these regions was assessed by bacteriocin-binding assays and by analyzing bacteriocin sensitivity of strains carrying mutated RseP. The results shed light on the interaction between EntK1 and RseP, providing insights into bacteriocin specificity and giving valuable information for the design of novel bacteriocins.

## Results

### Heterologous expression of RseP renders insensitive *Lactiplantibacillus plantarum* sensitive to EntK1

*L. plantarum* WCFS1 is a Gram-positive bacterium for which pSIP-based vectors have been developed for heterologous protein expression (31, 32). In addition, the bacterium is insensitive to EntK1 despite having an *rseP* ortholog on the chromosome. Together, these properties make *L. plantarum* a suitable host for expressing *E. faecium* RseP for binding and sensitivity studies. As shown in Table 1, expression of RseP from *E. faecium* renders *L. plantarum* sensitive to EntK1, with a minimum inhibitory concentration (MIC_50_) of 0.01 μM, while *L. plantarum* carrying the empty vector (pEV) exhibited a MIC_50_ greater than 22 μM, which is considered fully resistant. We also overexpressed *L. plantarum* RseP (*Lp*RseP) in *L. plantarum*, to confirm the inability of *Lp*RseP to be a receptor for EntK1. As expected, the *Lp*RseP-overexpressing strain (LpRseP^-His^; see Table 2 for a description of strain names) remained insensitive (i.e., MIC_50_ greater than 22 μM) (Table 1). These results suggest that the *L. plantarum* strain is a suitable host for heterologous expression of *Efm*RseP. Moreover, a pairwise sequence alignment of *Efm*RseP and *Lp*RseP indicates that subtle sequence differences between *Efm*RseP and *Lp*RseP define the sensitivity toward EntK1 (Fig. S1).

**Table 1:** MIC for EntK1 and FITC-EntK1 towards *Lactiplantibacillus plantarum* strains expressing heterologous RseP

Strains	Characteristics	MIC_50_ (μM)	
		EntK1	FITC-EntK1
EfmRseP^-His^	Expressing RseP from *Enterococcus faecium*	0.01	0.15
LpRseP^-His^	Expressing RseP from *Lactiplantibacillus plantarum*	>22	>20
EfsRseP^-His^	Expressing RseP from *Enterococcus faecalis*	0.04	0.6
LlRseP^-His^	Expressing RseP from *Lactococcus lactis*	0.09	>20
ShRseP^-His^	Expressing RseP from *Staphylococcus haemolyticus*	0.17	>20
SaeRseP^-His^^a^	Expressing RseP from *Staphylococcus arlettae*	>22	>20
SasRseP^-His^^a^	Expressing RseP from *Staphylococcus aureus*	>22	>20
pEV	Empty vector	>22	>20

a Control experiments (Fig. S2) indicated low expression, which may contribute to low sensitivity.

**Table 2:** Plasmids and bacterial strains used in this study

Strain or plasmid	Relevant characteristic(s)	Reference
Plasmid		
pLp1261_InvS	Spp-based expression vector, pSIP401 backbone, Ery^R^	(31, 41)
Strain		
*L. plantarum* WCFS1 (*Lp*)	Template for *rseP* (*Lp*RseP) and expression host	(53)
*E. faecium* P21 (*Efm*)	Template for *rseP* (*Efm*RseP)	(54)
*E. faecalis* V583 (*Efs*)	Template for *rseP* (*Efs*RseP)	NCBI:txid226185
*L. lactis* IL1403 (*Ll*)	Template for *rseP* (*Ll*RseP)	NCBI:txid272623
*S. aureus* ATCC 14458 (*Sas*)	Template for *rseP* (*Sas*RseP)	Nofima
*S. arlettae* LMGT 4134 (*Sae*)	Template for *rseP* (*Sae*RseP)	LMGT
*S. haemolyticus* LMGT 4106 (*Sh*)	Template for *rseP* (*Sh*RseP)	LMGT
*E. coli* TOP10	Cloning host	Thermo Fisher Scientific
*L. plantarum* WCFS1	Harboring pSIP401 encoding various RseP derivates, Ery^R^	
pEV	Empty vector	(41)
EfmRseP^-His^	*rseP* from *E. faecium* P21	This study
EfmRseP^a^	*rseP* from *E. faecium* P21, C-terminal 6× His-tag	This study
EfsRseP^-His^	*rseP* from *E. faecalis* V583	This study
LlRseP^-His^	*rseP* from *L. lactis* IL1403	This study
LpRseP^-His^	*rseP* from *L. plantarum* WCFS1	This study
LpRseP^a^	*rseP* from *L. plantarum* WCFS1, C-terminal 6× His-tag	This study
ShRseP^-His^	*rseP from S. haemolyticus* 7067	(33)
SasRseP^-His^	*rseP* from *S. aureus* ATCC 14458	This study
SaeRseP^-His^	*rseP* from *S. arlettae* LMGT 4134	This study
EfmH18A^a^	*Efm*RseP with substitution H18A	This study
EfmE19A^a^	*Efm*RseP with substitution H19A	This study
EfmH22A^a^	*Efm*RseP with substitution H22A	This study
EfmAAxxA^a^	*Efm*RseP with substitutions H18A, H19A, H22A	This study
EfmN359A^a^	*Efm*RseP with substitution N359A	This study
EfmN364A^a^	*Efm*RseP with substitution N364A	This study
EfmP367A^a^	*Efm*RseP with substitution P367A	This study
EfmP369A^a^	*Efm*RseP with substitution P369A	This study
EfmD372A^a^	*Efm*RseP with substitution D372A	This study
Hyb1^a^	Fusion of *Lp*RseP (1–221) and *Efm*RseP (222–422)	This study
Hyb2^a^	Fusion of *Lp*RseP (1–328) and *Efm*RseP (329–422)	This study
Hyb3^a^	Fusion of *Efm*RseP (1–221) and *Lp*RseP (222–425)	This study
Hyb4^a^	Fusion of *Efm*RseP (1–325) and *Lp*RseP (326–425)	This study
Hyb5^a^	Fusion of *Efm*RseP (1–200) and *Lp*RseP (201–425)	This study
Hyb6^a^	Fusion of *Efm*RseP (1–170) and *Lp*RseP (171–425)	This study
Hyb7^a^	Fusion of *Efm*RseP (1–32) and *Lp*RseP (33–425)	This study
Hyb8^a^	Fusion of *Lp*RseP (1–171, 222–425) and *Efm*RseP (172–221)	This study
Hyb9^a^	Fusion of *Lp*RseP (1–201, 222–425) and *Efm*RseP (202–221)	This study
Hyb10^a^	Fusion of *Lp*RseP (1–171, 326–425) and *Efm*RseP (172–325)	This study
Hyb11^a^	Fusion of *Lp*RseP (1–201, 328–425) and *Efm*RseP (202–327)	This study
Trunc^a^	Truncation of *Efm*RseP (1–39, 139–422) Δ40–138	This study

RseP homologs from the respective species are abbreviated with the species initials italicized (*e.g.*, *Sh*RseP is the RseP homolog in *S. haemolyticus*), while the strain names for each *L. plantarum* strain expressing a variant of RseP is not italicized (*e.g.*, ShRseP is *L. plantarum* WCFS1 harboring pSIP401 encoding *Sh*RseP). For cases where the species initials are ambiguous, both the first and last letter of the specific name is used (*e.g.*, *E*. *faecium* and *E*. *faecalis*).

Abbreviations: Em^R^, erythromycin resistance; LMGT, laboratory of microbial gene technology; Nofima, norwegian institute of food, fisheries and aquaculture research.

a Harboring a C-terminal 6× His-tag.

Of note, while the pSIP vectors used for expression (Table 2) have an inducible promoter, regulated by the inducer peptide SppIP (31), all sensitivity and binding experiments were performed using noninducing conditions. Under inducing conditions (3–30 ng/ml SppIP), the transformants showed aberrant growth on agar plates (data not shown), indicating a cytotoxic effect likely due to the high amounts of the membrane-protein RseP. Noninduced cells appeared to grow normally. The inducible promotor *sppA* in the pSIP vector has a low basal activity in *L. plantarum*, which permits low expression of *rseP* genes under noninducing conditions (as demonstrated by the results presented in Table 1).

### Antimicrobial activity and binding of EntK1 to sensitive cells depend on RseP

**Figure 2:**
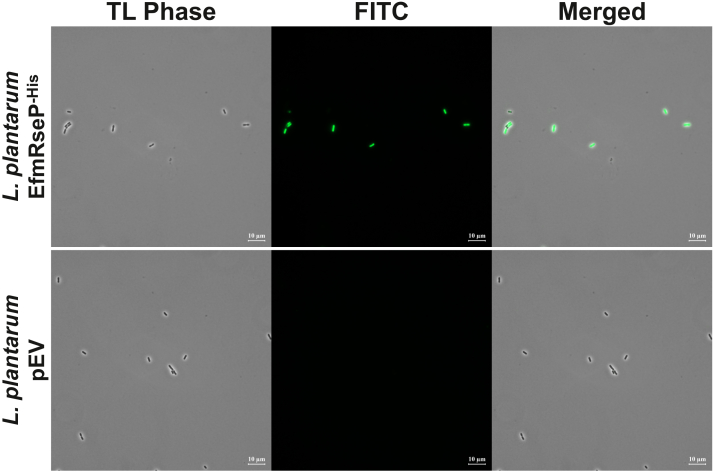
**Transmitted light phase contrast and fluorescence microscopy of *Lactiplantibacillus plantarum* expressing *Efm*RseP (EfmRseP**^**-His**^**) or containing the empty vector (pEV) after exposure to FITC-EntK1.** EfmRseP cells (*upper panel*) show strong fluorescent signals upon exposure to FITC-EntK1 compared to the negative control containing pEV (*lower panel*). An overlay of the fluorescence and phase-contrast images is shown to the right (Merged). *Efm*RseP, *Enterococcus faecium* RseP; EntK1, Enterocin K1.

To examine whether the antimicrobial activity observed above is directly linked to the ability of EntK1 to bind target cells, we developed a binding assay for EntK1 to *L. plantarum*. For this assay, EntK1 was chemically synthesized with an N-terminal FITC fluorescent tag. The N-terminal fusion was chosen as the C-terminal half of the LsbB family of bacteriocins and is thought to be necessary for receptor interaction (26). The labeling of EntK1 with FITC reduced the antimicrobial potency, which, however, remained high for *L. plantarum*–expressing *Efm*RseP (Table 1). Fluorescence microscopy of EntK1-sensitive *L. plantarum*–expressing plasmid-encoded *Efm*RseP showed strong fluorescent signals following exposure to FITC-EntK1, consistent with EntK1 binding. In contrast, nonsensitive *L. plantarum* carrying the empty vector (pEV) did not show any visible fluorescent signals under the same conditions, thus confirming lack of EntK1 binding (Fig. 2).

**Figure 3:**
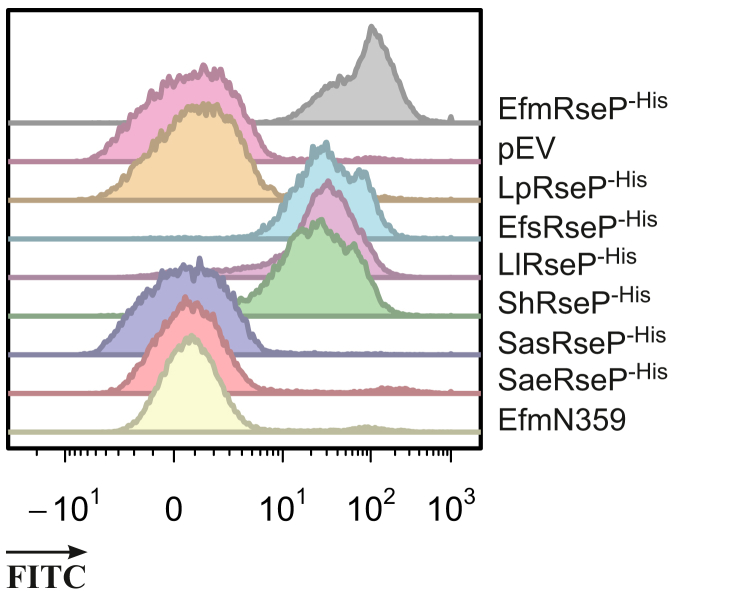
**FITC-EntK1 binding assay of *Lactiplantibacillus plantarum* clones expressing RseP from naturally EntK1-sensitive and EntK1-insensitive bacteria.** The figure shows representative histograms for *L. plantarum* cells expressing RseP from EntK1-sensitive species *Enterococcus faecium* (EfmRseP^-His^), *Enterococcus faecalis* (EfsRseP^-His^), *Lactococcus lactis* (LlRseP^-His^), and *Staphylococcus haemolyticus* (ShRseP^-His^), and from EntK1-insensitive species *L. plantarum* (LpRseP^-His^), *Staphylococcus arlettae* (SaeRseP^-His^), and *Staphylococcus aureus* (SasRseP^-His^), in addition to *L. plantarum* carrying the empty vector and the N359A mutant of *Efm*RseP. An increase in fluorescence indicates binding of the bacteriocin to the cells. EntK1, Enterocin K1.

In accordance with the fluorescence microscopy, flow cytometry analysis revealed that FITC-EntK1–exposed *L. plantarum*–expressing RseP derived from *E. faecium* exhibited strong fluorescent signals, while cells containing the empty vector or overexpressing the *Lp*RseP protein showed no signal (Fig. 3). We have previously observed that EntK1 has some antimicrobial activity toward strains of *L. lactis*, *E. faecalis*, and *Staphylococcus haemolyticus* but not strains of *Staphylococcus aureus* and *Staphylococcus arlettae* (33). To confirm that the sensitivity is linked to RseP binding, *rseP* genes derived from these sensitive and insensitive species were heterologously expressed in *L. plantarum*. Table 1 shows that, indeed, *L. plantarum* strains expressing *rseP* genes derived from the sensitive strains of *L. lactis* (LlRseP^-His^), *E. faecalis* (EfsRseP^-His^), and *S. haemolyticus* (ShRseP^-His^) were indeed inhibited by EntK1. In addition, Figure 3 shows that these strains had distinctly higher FITC signals than *L. plantarum* strains expressing RseP from the insensitive strains *S. aureus* (SasRseP^-His^) and *S. arlettae* (SaeRseP^-His^). Taken together, these results provide strong evidence that there is a specific interaction between EntK1 and RseP from bacteria that are naturally sensitive to EntK1 but not between EntK1 and RseP from bacteria that are insensitive to EntK1.

### Defining the role of conserved S2P motifs in the EntK1:RseP interaction

To define the regions of RseP involved in EntK1 sensitivity, we initially focused on conserved regions that, based on previous studies of other members of the S2P family, seem to be involved in substrate binding and catalysis. In addition to the conserved residues of the active site found in all members of the S2P family, *E. faecium* RseP contain multiple other conserved motifs, including the MRE β-loop and the extended LDG motif. These domains are conserved among members of subgroup I and III in the S2P family, as well as the GxG motif and PDZ domain which are only present in subgroup I (Fig. 1). To examine how these conserved motifs of *E. faecium* RseP affect the binding of and sensitivity toward EntK1, mutational analysis of each motif was performed, by site-directed mutagenesis, by creating hybrids of *Efm*RseP and *Lp*RseP, and by a truncation in *Efm*RseP.

#### The active site

The conserved motifs HExxH and LDG make up the active site of the S2P family (Fig. 1) (14). It has previously been shown that mutations of residues corresponding to *Efm*RseP His18, Glu19, His22, and Asp372 substantially affect the protease activity of RseP homologs from multiple species (12, 14, 34). To examine whether proteolytic activity of RseP is needed for EntK1 sensitivity, alanine substitutions were introduced in all conserved residues in the active site. Single alanine substitutions in the active site (EfmH18A, EfmE19A, EfmH22A, and EfmD372A) resulted in a slight increase of the MIC_50_ from ≤0.002 μM for 6His-tagged WT *Efm*RseP to 0.01 to 0.7 μM for the 6His-tagged mutants (Table 3). In line with these observations, measurements of the populations with the single alanine substitutions in the binding assay described above showed only a slight reduction in binding with 59 to 80% of the median fluorescence intensity of the *L. plantarum* population expressing the native *Efm*RseP. The triple alanine substitution (EfmAAxxA) resulted in a considerable increase in the MIC_50_, to 2.7 μM (Table 3). However, the triple mutant was still more than 8-fold more sensitive to EntK1 than pEV. The impact of the mutations on the MIC_50_ values could be partly due to variation in RseP expression, which was not assessed in detail. For example, it is conceivable that the triple mutant is rather unstable and was produced in lower amounts, leading to a higher MIC_50_ value and low EntK1 binding. Nevertheless, the fact that all variants remained sensitive and bound the bacteriocin clearly shows that the mutant proteins were produced and that the catalytic activity of RseP does not play an essential role in RseP binding and strain sensitivity.

**Table 3:** EntK1 sensitivity and EntK1 binding of *Lactiplantibacillus plantarum* expressing variants of RseP

Strain^a^	EntK1 MIC_50_ (μM)	FITC-EntK1 rMFI % (RSD)
EfmRseP	≤0.002	100 (8.3)
pEV	>22	0.4 (4.2)
LpRseP	>22	0.41 (9.7)
Active site		
EfmH18A	0.02	61.6 (3.8)
EfmE19A	0.01	65.4 (3.3)
EfmH22A	0.01	59 (8.1)
EfmAAxxA	2.7	0.5 (5.2)
EfmD372A	0.7	80.6 (3.5)
GxG motif and the MRE β-loop		
Trunc	2.7	7.4 (12.2)
Extended LDG		
EfmN359A	>22	0.41 (22.6)
EfmN364A	0.004	83.4 (3.4)
EfmP367A	0.004	89.1 (4.6)
EfmP369A	≤0.02	84.4 (6.9)
RseP hybrids		
Hyb1	>22	0.42 (25.2)
Hyb2	>22	0.47 (18.3)
Hyb3	0.7	37.6 (24.4)
Hyb4	≤0.002	97.6 (8.5)
Hyb5	>22	0.82 (36.6)
Hyb6	>22	0.51 (6.8)
Hyb7^b^	>22	0.29 (10.4)
Hyb8	>22	0.82 (26.6)
Hyb9	>22	0.53 (18.2)
Hyb10	≤0.002	78 (7.6)
Hyb11	0.09	54.8 (2.4)

The middle column shows MIC for EntK1 towards *L. plantarum* strains expressing various RseP variants (see text, Figs. 1 and S1 for details). The strains are named by the protein variant they express. The right column shows the binding of FITC-labeled EntK1 to indicated strains. The FITC signals, indicating binding of the bacteriocin, are presented as the relative median fluorescence intensity (rMFI) compared to the MFI obtained for EfmRseP6His (100%) with percent relative standard deviations (RSD).

a All RseP variants contain a C-terminal 6× His-tag.

b Control experiments (Fig. S2) indicated low expression, which may contribute to low sensitivity.

#### The MRE β-loop and the GxG motif region

Previous studies on *Ec*RseP indicate that the MRE β-loop and the GxG motif region (Fig. 1) interact directly with the substrate (20, 22). To examine the significance of this region for the RseP:EntK1 interaction, residues 39 to 138 encompassing the MRE β-loop and the GxG motif were deleted (Trunc, Fig. S1). The truncation significantly reduced EntK1 sensitivity, as judged by the increase in MIC_50_ of Trunc to 2.7 μM (Table 3). Using the binding assay, we observed that FITC signals were also significantly reduced to 7.4% compared to the full-length protein (Table 3). Nonetheless, the FITC signal reflecting binding (7.4% *versus* 0.4%) and the sensitivity towards EntK1 (MIC_50_ of 2.7 μM *versus* 22 μM) were higher than that of the empty vector control strain (Table 3). It would thus seem that the MRE β-loop and the GxG motif region are not involved in the RseP:EntK1 interaction.

#### The extended LDG motif

A conserved motif in TMS3 (NxxPxPxLDG), which includes the LDG catalytic site motif (Fig. 1*B*), has been suggested as a prime candidate for S2P substrate binding (13). Moreover, previous substrate-binding studies with *Ec*RseP (24) suggest a longer version of the LDG motif, referred as the extended LDG motif (N359xxxxN364xxP367xP369xLD372G in *Efm*RseP), may be important for substrate binding. The two asparagines and two prolines in the extended motif were individually mutated to alanine. Three of the four mutants remained sensitive to EntK1 and showed strong EntK1 binding (Table 3). However, the alanine substitution of Asn359 in *Efm*RseP (named EfmN359A) resulted in complete resistance to EntK1, and the binding of the bacteriocin was abolished (Fig. 3 and Table 3).

The absence of EntK1 sensitivity and EntK1 binding could be caused by failure to express the *rseP* variant. Therefore, EfmN359A (and all other variants displaying a complete loss of sensitivity, discussed below) were exposed to EntEJ97, another bacteriocin from the LsbB family. EntEJ97 targets RseP but has a different antimicrobial spectrum compared to EntK1 (11), which implies that its interaction with RseP differs from EntK1. Fig. S2 shows that the control pEV clone displayed limited sensitivity towards EntEJ97, while EfmN359A was highly sensitive to the bacteriocin, showing that the alanine substitution did not drastically alter the protein structure nor the expression level and that the removal of the asparagine side chain alone is likely responsible for the alteration in EntK1 sensitivity and binding. Interestingly, Asn359 and the extended LDG motif are highly conserved among both EntK1-sensitive and EntK1-insensitive species (Fig. 1*B*). Thus, the impact of this residue on EntK1 sensitivity must relate to its interaction with other less conserved regions of the protein.

### Mapping the regions involved in EntK1 specificity.

To further identify regions determining EntK1 sensitivity, we constructed several hybrid proteins in which parts of the RseP from insensitive *L. plantarum* were replaced with the corresponding parts of RseP from sensitive *E. faecium* (Hyb1-11, Fig. S3). Previous studies had suggested that residues 328 to 428 in the C-terminal region of RseP from *L. lactis* (YvjB) determine the sensitivity of *L. lactis* to LsbB (35). As LsbB and EntK1 target the same receptor, belong to the same bacteriocin family, and have a similar structure (10, 11, 26), we hypothesized that the C-terminal region of RseP from *E. faecium* would confer EntK1 sensitivity. To test the hypothesis, varying parts of the C-terminal region of *Lp*RseP were replaced with the corresponding region of *E. faecium* RseP (Fig. S3). Surprisingly, the resulting hybrid proteins, Hyb1 and Hyb2, did not confer sensitivity to EntK1 (MIC_50_ > 22 μM) nor did they show bacteriocin binding (Table 3). Control experiments with EntEJ97 (Fig. S2) showed that Hyb1 and Hyb2 were produced.

Next, Hyb3 and Hyb4 (inverts of Hyb1 and Hyb2), containing the N-terminal region of *Efm*RseP and the C-terminal region of *Lp*RseP were constructed (Fig. S3). Unlike Hyb1 and Hyb2, Hyb3 and Hyb4 conferred sensitivity to EntK1 with MIC_50_ values of 0.7 μM and ≤0.002 μM, respectively. Hyb3 and Hyb4 also showed binding of the bacteriocin (Table 3). The results obtained with Hyb1-4 show that the N-terminal region of *E. faecium* RseP (residues 1–324) is involved in EntK1 binding. Although quantitative comparison of MIC_50_ values is risky due to possible differences in expression, it is worth noting that Hyb4, containing the complete *Efm*RseP PDZ domain, was the most sensitive of the four hybrids.

To further narrow down the RseP region needed for EntK1 sensitivity, three additional hybrid proteins containing a decreasing portion of *Efm*RseP were constructed (Hyb5-7, Fig. S3). None of these hybrids, all lacking the PDZ domain from *E. faecium*, could confer sensitivity to or binding of EntK1 (Table 3), indicating that the PDZ domain is required for activity. The control experiments of Fig. S2 showed that Hyb5 and Hyb6 were produced, whereas Hyb7 likely has reduced expression. To confirm the importance of the PDZ region, we constructed four additional hybrid proteins in which different parts of the PDZ domain of *Lp*RseP were replaced with the corresponding sequences of *Efm*RseP (Fig. 3, Hyb8-11). Interestingly, only Hyb10 and Hyb11, which contained the entire PDZ domain from *Efm*RseP were EntK1-sensitive, with MIC_50_ values of 0.002 μM and 0.09 μM, respectively (Table 3). Hyb8 and Hyb9, only containing parts of the *Efm*RseP PDZ domain, were not sensitive to EntK1 with MIC_50_ >22 μM (Table 3). A control experiment showed that both Hyb8 and Hyb9 were highly sensitive to EntEJ97, indicating that these hybrids are produced (Fig. S2).

Importantly, as noted above, all hybrids that did not confer sensitivity or binding to EntK1, except for Hyb7, were sensitive (*i.e.*, inhibition zone >10 mm for EntEJ97; Fig. S2). This indicates that Hyb1-6 and Hyb8-11 were properly expressed and folded. Moreover, all clones of *L. plantarum*–expressing recombinant RseP showed growth comparable to EfmRseP, suggesting that expression of the hybrids had no obvious toxic effect on the host (data not shown).

## Discussion

The S2P RseP is highly conserved in multiple species, yet the potency of EntK1 varies considerably between species (11, 33). To further develop EntK1 as a novel treatment option for bacterial infections, a detailed understanding of the determinants of bacteriocin sensitivity and binding to RseP is essential. Therefore, in this study, we explored the contribution of conserved S2P motifs to the EntK1:RseP interaction and EntK1 sensitivity. To do so, we first needed to establish a sensitivity and binding assay. Although the antimicrobial activity of EntK1 depends on RseP (11), it remains elusive whether the difference in EntK1 sensitivity between species is solely due to variations in the RseP protein or if other factors, such as cell surface composition and gene expression levels, contribute. To avoid potential problems related to these uncertainties, we expressed *rseP* from insensitive and sensitive species in the same expression vector (pSIP) and EntK1-insensitive host (*L. plantarum*). We demonstrated that only *rseP* from sensitive bacterial species confers EntK1 sensitivity to *L. plantarum*. Binding of the bacteriocin to the RseP-producing *L. plantarum* strains was assessed using FITC-labeled EntK1. The levels of FITC-EntK1 signals correlated well with the MIC_50_ values (Table 3; higher binding correlates with lower MIC_50_ values). These observations show that subtle differences in the receptor alone likely determine variation in EntK1 sensitivity.

*Efm*RseP belongs to group I of the S2P family, for which the involvement of several conserved motifs in substrate binding and substrate specificity has been explored to some extent (20, 22, 24, 34). We considered that these motifs could be involved in EntK1 sensitivity and, therefore, we targeted these motifs in the mutagenesis studies to identify their role(s) in RseP as a bacteriocin receptor. We initially focused on the active site of *E. faecium* RseP, as there were indications in the literature that alterations in the active site of RseP in *E. faecalis* affects the sensitivity to a member of the LsbB bacteriocin family (11). However, none of the mutations in the catalytic center, including mutations known to abolish protease activity in *Ec*RseP (16, 36), led to EntK1 resistance, demonstrating that the proteolytic activity of RseP is not essential for interaction and the antimicrobial action of EntK1.

Two motifs of *Ec*RseP known to interact with the substrate are the MRE β-loop and a conserved GxG motif located on a membrane-associated region between TMS1 and TMS2 (Fig. 1) (20, 22). If RseP-targeting bacteriocins mimic the interaction of natural substrates with the receptor, these two regions would likely interact with EntK1. Although deletion of the MRE β-loop and the GxG motif led to a significant reduction in EntK1 sensitivity, the removal of these nearly 100 amino acids did not result in total resistance toward EntK1 (Table 3), indicating that neither the MRE β-loop nor the GxG motif is essential for the EntK1:RseP interaction. The reduced sensitivity and binding upon truncation are likely due to global structural changes in the receptor resulting from the large deletion. Of note, the MRE β-loop and GxG motif of *Efm*RseP are both predicted to be located on the cytoplasmic side of the cell membrane (Fig. 1*A*); such a location would likely not allow direct interaction with the bacteriocin which attacks target cells from the outside. It should be noted that the predicted topology of *Efm*RseP and the RseP hybrids was not confirmed experimentally in this study. However, a similar topology for the group 1 S2P *Ec*RseP and *Sas*RseP has been confirmed by the fusion of alkaline phosphatase to specific regions of RseP (6, 15), suggesting that the predicted topology may be conserved among group 1 S2Ps.

Next, we explored the role of the extended LDG motif in EntK1:RseP interaction. Substituting Asn364, Pro367, and Pro369 with alanine in *Efm*RseP resulted in only minor changes in EntK1 sensitivity and binding (Table 3). This was surprising, as these conserved asparagine and proline residues are known to be important for substrate binding and correct processing in both *Ec*RseP and S2P from *B. subtilis*, known as SpoIVFB (12, 24, 37, 38). On the other hand, Asn359 was shown to be essential for EntK1 sensitivity and binding (Table 3). Under noninduced conditions, we were not able to detect RseP expression from N359A, or any other clone, using a standard Western blot (Fig. S4). However, when induced, expression levels of N359A and *Efm*RseP were comparable, suggesting that the insensitivity of the clone was due to the N359A substitution but not due to a failure in expression. Moreover, EfmN359A remained highly sensitive to EntEJ97, another bacteriocin of the LsbB family targeting RseP, which strongly indicates that the observed changes in sensitivity and binding were not caused by failure to express mutated *rseP* (Fig. S2). Previous studies have exploited the known substrates of RseP homologs to perform cleavage-based activity assays to confirm proper protein folding and expression following the introduction of mutations (24). However, RseP has no known substrates in *E. faecium*, which explains why cleavage-based activity assays could not be used. Interestingly, Asn389 in *Ec*RseP, which corresponds to Asn359 in *Efm*RseP, plays an important role in substrate recognition. When this asparagine was replaced by cysteine, *Ec*RseP showed reduced substrate binding as well as reduced proteolytic activity (24). Despite the evident role of Asn359 in EntK1:RseP binding, it is interesting to note that Asn359 and the surrounding extended LDG domain are highly conserved in the RseP proteins of both EntK1-sensitive and EntK1-insensitive species (Fig. 1*B*). This suggests that other regions of RseP play a role in bacteriocin binding and sensitivity.

Of the 11 constructed *Efm*RseP-*Lp*RseP hybrid proteins, only four (Hyb3, Hyb4, Hyb10, Hyb11) were EntK1 sensitive (Table 3). Importantly, all EntK1-sensitive hybrids contain parts of the *Efm*PDZ domain, with Hyb4, Hyb10, and Hyb11 containing the entire domain. Of the four sensitive hybrids, hybrids containing the entire *Efm*PDZ domain exhibited the lowest MIC_50_ (*i.e.*, most sensitive), underpinning the important contribution of this domain to the EntK1:RseP interaction. Previous studies have shown that the PDZ domain is involved in substrate recognition by RseP-like S2P (21, 23). It has been suggested that the PDZ domain of *Ec*RseP acts as a size-exclusion filter, preventing substrates with large periplasmic domains access to the active site (21). A similar role has been suggested for the PDZ domain of the *B. subtilis* S2P homolog, RasP (23). Several S2Ps process multiple substrates *in vitro* and *in vivo*, yet the substrate specificity of these proteins is poorly understood. We conclude that the PDZ domain of *Efm*RseP is the defining region for EntK1 binding and thus the major determinant of variation in EntK1 sensitivity.

**Figure 4:**
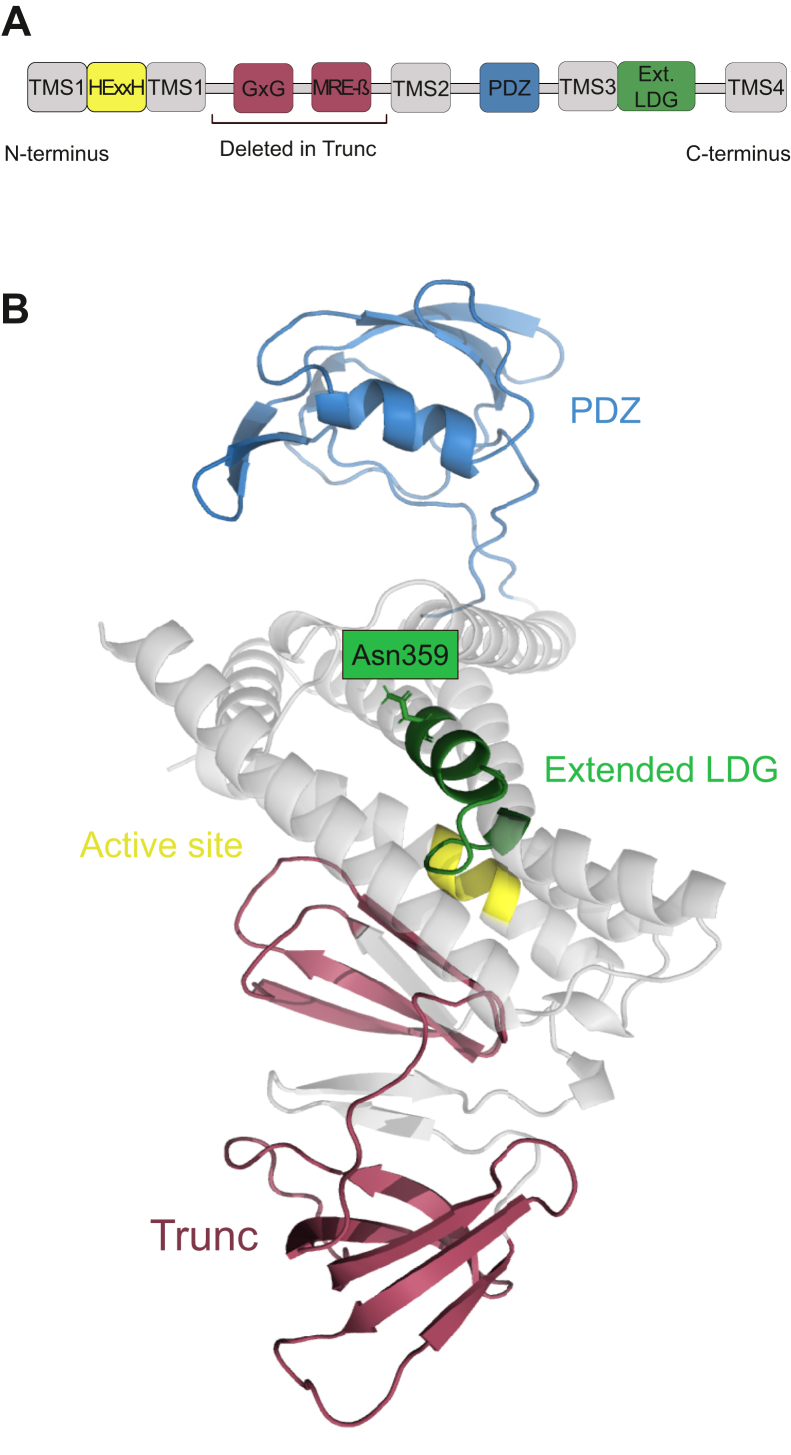
**RseP from *Enterococcus faecium* as predicted by AlphaFold.***A*, schematic overview of the conserved RseP-like S2P motifs found in RseP from *E. faecium*. *B*, structure of RseP from *E. faecium* as predicted by AlphaFold with RseP-like S2P motifs highlighted. The HExxH motif of the active site is indicated in *yellow*, the predicted PDZ domain is indicated in *blue*, and the extended LDG domain is indicated in *green*. The region deletion from Trunc, which encompasses the GxG and the MRE β-loop motifs, is indicated in *purple*. MRE β-loop, membrane-reentrant β-hairpin–like loop; S2P, site-2-metalloprotease.

To better understand the positions of *Efm*RseP motifs investigated in this study, we predicted the structure of *Efm*RseP using AlphaFold. AlphaFold is a protein structure prediction program based on artificial intelligence that predict protein structures with greater accuracy than any other in silico method (39). As illustrated in Figure 4, AlphaFold predicted that the PDZ domain of *Efm*RseP forms a pocket which may prevent direct access to core residues. Among the regions investigated in this study, Asn359 in the extended LDG domain is located the closest to the PDZ domain, while the GxG motif and the MRE-β loop appears to be more distant (Fig. 4). Taken together with our experimental data, it is conceivable that the initial docking of EntK1 to the PDZ domain leads to subsequent interactions with core residues such as Asn359.

During the finalization of this article, AlphaFold-Multimer was published (R. Evans *et al*., Preprint at bioRxiv). AlphaFold-Multimer is an extension of AlphaFold2 using an artificial intelligence model explicitly trained for multimeric input. This allowed us to predict the EntK1:RseP complex, which strikingly predicted the interaction between EntK1 and RseP to primarily involve the PDZ domain and the region near Asn359 (data not shown). However, while most of the residues of RseP in the complex exhibited a high confidence score (pLDDT > 90), most of the residues of EntK1 were ranked poorly (pLDDT < 50). Confidence scores below 50 is a strong predictor of disorder, suggesting that the peptide chain is unstructured at physiological conditions or only structured as part of a complex. Indeed, EntK1 has been shown to be disordered in an aqueous environment by NMR spectroscopy (11). Due to the low confidence scores produced for EntK1 in the complex, these structure predictions are highly speculative and should be used cautiously.

While it remains unknown how the EntK1:RseP complex eventually leads to cell death, the present study reveals molecular details of the interaction of EntK1 with its receptor. Previous studies have shown that bacteriocins of the LsbB family can be engineered to improve both potency and alter the activity spectrum (33). The interpretation of these previous results, as well as future efforts to develop improved RseP-binding bacteriocins, will benefit from the deeper insight into the bacteriocin–receptor interaction that we provide here. Importantly, LsbB family of bacteriocins are attractive not only because they act on vancomycin-resistant strains but also because the bacteriocins are short, synthesized without an N-terminal leader sequence, and contain no posttranslational modification, which enables low-cost synthetic production. The fact that RseP homologs have important roles in virulence in several animal and human pathogens highlights RseP as an attractive antimicrobial target in multiple species (9, 40). The mutational analysis performed in this study combined with the predicted *Efm*RseP structure may provide a powerful basis for guided construction of novel bacteriocins and may contribute to further development of RseP as a drug target.

## Experimental procedures

### Bacterial strains and cultivation conditions

Bacterial strains used in this study are listed in Table 2. The following strains were cultivated in Brain heart infusion broth (Thermo Scientific Oxoid ): enterococcal strains (37 °C, without agitation), staphylococcal strains (37 °C, 220 rpm), and *E. coli* (37 °C, 220 rpm). *L. plantarum* and *L. lactis* were cultivated without shaking in DeMan, Rogosa and Sharp (MRS) broth (Thermo Scientific Oxoid ) at 37 °C and M17 broth (Thermo Scientific Oxoid ) supplemented with 0.5% glucose at 30 °C, respectively. Agar plates were prepared by supplementing the appropriate broth with 1.5% (w/v) agar (VWR chemicals). Erythromycin was added to a final concentration of 200 μg/ml for *E. coli* and 10 μg/ml for *L. plantarum* when appropriate.

### Construction of *rseP* orthologs, *rseP* hybrids, and site-directed mutagenesis

Seven orthologs of *rseP* from EntK1-sensitive and EntK1-insensitive species were expressed in *L. plantarum* using the pSIP expression system (31, 32) (Table 2). Briefly, pLp1261_InvS, a pSIP derivative, was digested with NdeI and Acc65I or XmaI (Thermo Fisher Scientific) (41). Genomic DNA from the seven native *rseP*-containing strains was used as a template for the amplification of *rseP*. PCR amplification of all *rseP* variants was performed using Q5 Hot Start High-fidelity DNA polymerase (New England BioLabs) with In-Fusion primers to yield amplicons with ends complementary to the linearized pSIP vector (Table S1). The amplified PCR fragments were fused with the linearized vector using In-Fusion HD cloning Kit (Takara Bio) and transformed into competent *E. coli* TOP10 (ThermoFisher Scientific).

Site-directed mutants, truncations of RseP and RseP hybrids were constructed using splicing by overlap extension PCR. Briefly, two fragments of the *rseP* sequences were amplified in separate PCR reactions using two primer pairs, each consisting of an inner and outer primer (Table S1). The inner primers generated overlapping complementary ends and acted as mutagenic primers when introducing point-mutations. The overlapping fragments were fused by a second PCR reaction using the outer primers. Fused amplicons containing a mutated *rseP* gene were purified, fused to the linearized vector, and transformed to *E. coli* as described above.

All constructed plasmids were verified by DNA sequencing at Eurofins GATC Biotech (Germany) and subsequently transferred into electrocompetent *L. plantarum* as previously described (42). Fig. S3 shows a schematic representation of all hybrids and the truncated versions of RseP. Protein topology and the PDZ domain were predicted using CCTOP and Pfam, respectively (43, 44).

### Antimicrobial assays

The bacteriocins used in this study, EntK1, EntEJ97, and FITC-EntK1, were produced by Pepmic Co, LtD with >95% purity. Bacteriocins were solubilized in 0.1% (vol/vol) TFA (Sigma-Aldrich), except for FITC-EntK1 which was solubilized in dH_2_O. For semiquantitative assessment of antimicrobial activity, a spot-on-lawn assay was performed. Briefly, an overnight culture was diluted 1:100 in soft-agar and distributed on agar plates containing appropriate antibiotics. Bacteriocins with various concentrations were applied on designated spots on the solidified soft-agar. The agar plates were incubated at appropriate temperatures overnight and inhibition zones were measured the following day. For more accurate quantification, EntK1 sensitivity was determined using a microtiter plate assay to define MIC_50_ (45). The MIC_50_ was defined as the lowest bacteriocin concentration needed to inhibit bacterial growth by ≥50%. MIC assays were performed with three biological replicates.

### Binding assays

Overnight cultures of *L. plantarum* strains were diluted 50-fold and grown until mid-log phase (4 h), after which cells were harvested by centrifugation at 16,000*g* for 3 min and resuspended in sterile 0.9 % (w/v) NaCl to an A_600_ of 1 (assessed using a SPECTROstar Nano reader; BMG Labtech). Cell suspensions were diluted 20-fold in binding buffer [1 μM FITC-labeled EntK1 in 100 μM triammonium citrate pH 6.5 (Sigma-Aldrich)]. The cells were incubated in the binding buffer on a rotator (Multi Bio RS-24, Biosan, Latvia) at 6 rpm for 20 min at room temperature. After incubation, cells were harvested by centrifugation (16,000*g*, 3 min) and the binding buffer was discarded. The cell pellets were resuspended in sterile PBS to an appropriate cell density and analyzed using a MACSQuant Analyzer flow cytometer with excitation at 488 nm and emission at 475 to 575 nm (500 V PMT). The instrument was set to trigger on side-scattered light (SSC-A, 370 V PMT) with the threshold set to 8 to reduce false events.

Data and figures were prepared using the CytoExploreR package (v 1.1.0) for the R programming language (v 4.0.5) (https://github.com/DillonHammill/CytoExploreR [accessed June 25, 2022], https://www.R-project.org/ [accessed June 28, 2022]). All binding assays were performed in triplicate. The median fluorescence intensity (MFI) was calculated as the average of three runs for each strain and expressed as a percent relative to *L. plantarum* expressing RseP from *E. faecium* (rMFI). Percent relative standard deviations were calculated as the ratio of the sample SD to the MFI mean multiplied by 100%.

### Phase contrast and fluorescence microscopy

The cells were stained with the FITC-labeled EntK1 as described for the binding assay. After discarding the remaining binding buffer, cells were resuspended in 25 μl of PBS, spotted on a microscopy slide, and overlayed with 2% low melting agarose in PBS to immobilize the cells. Phase-contrast images and FITC fluorescence images were obtained using a Zeiss Axio Observer with ZEN Blue software and an ORCA-Flash 4.0 V2 Digital CMOS camera (Hamamatsu Photonics) using a 100 × phase-contrast objective. The excitation light source was an HXP 120 Illuminator (Zeiss).

### AlphaFold and structure analysis

The structure of RseP and complexes between RseP and EntK1 were predicted using the published open source code for AlphaFold according to the instructions by the AlphaFold team (46). All required databases were downloaded on February 10th 2022 and all templates prior to 2022 were included (--max_template_date = 2022-01-01). Interactions present in the predicted complexes were determined by the fully automated protein-ligand interactions profiler (47) and the interactions function implemented in the web-based molecular viewer iCn3D (48, 49). Figures were generated using PyMOL (http://www.pymol.org/pymol). Amino acid sequences used for *Efm*RseP and EntK1 are presented in Table S2.

## Data availability

The AlphaFold computations were performed on resources provided by Sigma2 (allocations NN1003K and NS1003K) - the National Infrastructure for High Performance Computing and Data Storage in Norway. For DNA sequence of the mutants and flow cytometry, the data will be shared upon request.

## Supporting information

This article contains supporting information (50, 51, 52).

## Conflict of interest

The authors declare that they have no conflicts of interest with the contents of this article.

## Supporting information

Supplemental Tables

Figure S1 **Pairwise sequence alignment of *Efm*RseP and *Lp*RseP.** Identical amino acids are shaded in black while amino acids of similar chemical properties are boxed. Alignment was generated using EMBOSS Needle and the figure was exported using ESPript 3 web-server (51, 52).

Figure S2 **EntEJ97 inhibition zones in spot-on-lawn assays.** 3 μl of EntEJ97 and EntK1 (4–220 μM) was spotted on lawns of *L. plantarum* clones expressing different modified versions of RseP. Since RseP is the receptor for EntEJ97, increased sensitivity towards EntEJ97 compared to the empty vector pEV may indicate proper production and folding of the target protein expression. An inhibition zone of >10 mm was observed for all hybrids and point mutants tested, except for Hyb7, SaeRseP, SasRseP and pEV. A zone comparable to pEV was observed for Hyb7, SaeRseP and SasRseP (<6 mm).

Figure S3A **Schematic representation of all RseP hybrids and truncated RseP.** Hybrid proteins in which parts of *Lp*RseP (from the EntK1-insensitive *L. plantarum*, indicated in *purple*) were replaced with the corresponding sequence from *Efm*RseP (from the EntK1-sensitive *E. faecium*, indicated in *green*), were constructed. Conserved S2P motifs are indicated (HExxH, GxG, MRE-ß, PDZ, LDG). TMS1-4 indicate the four predicted transmembrane segments (TMS), while the black arrows indicate the point(s) of fusion between *Lp*RseP and *Efm*RseP regions. The gray dashed line indicates part of the full-length *Efm*RseP deleted in Trunc. Protein variants giving a MIC_50_ ≥22 μM EntK1 are marked as fully EntK1-resistant, while protein variants giving MIC_50_ values below 2.7 μM are marked as EntK1-sensitive. Note that sensitivity data for Hyb7 are uncertain due to possible expression issues visible in the control experiment of Fig. S2. For MIC_50_ values, see Table 3.

Figure S3B

Figure S4 **Western blot.** pEV, EfmRseP and the N359A mutant under inducing (+) and non-inducing (-) conditions. Cells were harvested, lysed and subjected to SDS-PAGE as previously described (50), with minor modifications. The harvested cells were resuspended in 250 μl NP-40 lysis buffer containing 1 mM PMSF (phenylmethylsulfonyl fluoride). Samples where not boiled prior to SDS-PAGE. Following electrophoresis, proteins were electroblotted onto nitrocellulose mini membranes using iBlot^TM^ Transfer Stack (Invitrogen) and the iBLot Gel transfer device (Invitrogen). The membrane was washed with Tris-Buffered Saline (TBS; 2 × 10 min), then subsequently incubated with blocking buffer (5% Bovine Serum Albumin (BSA) dissolved in TBS) for 1 h and washed again (2 × 10 min Tween-TBS (TTBS; 0.05% Tween-20), 1 × 10 min TBS). The membrane was incubated with Penta-His (Qiagen) (1:1000 in Blocking buffer) for 30 min, following an overnight incubation at 4 °C. The subsequent day the membrane was incubated for 30 min at room temperature, washed (2 × 10 min, TTBS), and incubated with the polyclonal HRP-conjugated anti-mouse IgG (Sigma-Aldrich) secondary antibody (1:5000) in blocking buffer for 1 h. To remove unbound secondary antibodies the membrane was washed 4 × 10 min with TTBS. The blots were subsequently visualized using the SuperSignal West Pico PLUS Chemiluminescent substrate (Thermo Fisher Scientific) and image using the Azure c400 system (Azure Biosystem, Dublin, CA).

## References

[1] Brown M.S., Ye J., Rawson R.B., Goldstein J.L. (2000). Regulated intramembrane proteolysis: a control mechanism conserved from bacteria to humans. Cell.

[2] Kroos L., Akiyama Y. (2013). Biochemical and structural insights into intramembrane metalloprotease mechanisms. Biochim. Biophys. Acta.

[3] Chen J.C., Viollier P.H., Shapiro L. (2005). A membrane metalloprotease participates in the sequential degradation of a Caulobacter polarity determinant. Mol. Microbiol..

[4] Yokoyama T., Niinae T., Tsumagari K., Imami K., Ishihama Y., Hizukuri Y. (2021). The *Escherichia coli* S2P intramembrane protease RseP regulates ferric citrate uptake by cleaving the sigma factor regulator FecR. J. Biol. Chem..

[5] King-Lyons N.D., Smith K.F., Connell T.D. (2007). Expression of hurP, a gene encoding a prospective site 2 protease, is essential for heme-dependent induction of bhuR in *Bordetella bronchiseptica*. J. Bacteriol..

[6] Cheng D., Lv H., Yao Y., Cheng S., Huang Q., Wang H. (2020). The roles of the site-2 protease Eep in *Staphylococcus aureus*. J. Bacteriol..

[7] Frank K.L., Barnes A.M., Grindle S.M., Manias D.A., Schlievert P.M., Dunny G.M. (2012). Use of recombinase-based *in vivo* expression technology to characterize *Enterococcus faecalis* gene expression during infection identifies in vivo-expressed antisense RNAs and implicates the protease Eep in pathogenesis. Infect. Immun..

[8] Schöbel S., Zellmeier S., Schumann W., Wiegert T. (2004). The *Bacillus subtilis* sigmaW anti-sigma factor RsiW is degraded by intramembrane proteolysis through YluC. Mol. Microbiol..

[9] Schneider J.S., Glickman M.S. (2013). Function of site-2 proteases in bacteria and bacterial pathogens. Biochim. Biophys. Acta Biomembr..

[10] Uzelac G., Kojic M., Lozo J., Aleksandrzak-Piekarczyk T., Gabrielsen C., Kristensen T. (2013). A Zn-dependent metallopeptidase is responsible for sensitivity to LsbB, a class II leaderless bacteriocin of *Lactococcus lactis* subsp. lactis BGMN1-5. J. Bacteriol..

[11] Ovchinnikov K.V., Kristiansen P.E., Straume D., Jensen M.S., Aleksandrzak-Piekarczyk T., Nes I.F. (2017). The leaderless bacteriocin enterocin K1 is highly potent against *Enterococcus faecium*: a study on structure, target spectrum and receptor. Front Microbiol..

[12] Rudner D.Z., Fawcett P., Losick R. (1999). A family of membrane-embedded metalloproteases involved in regulated proteolysis of membrane-associated transcription factors. Proc. Natl. Acad. Sci. U. S. A..

[13] Kinch L.N., Ginalski K., Grishin N.V. (2006). Site-2 protease regulated intramembrane proteolysis: sequence homologs suggest an ancient signaling cascade. Protein Sci..

[14] Feng L., Yan H., Wu Z., Yan N., Wang Z., Jeffrey P.D. (2007). Structure of a site-2 protease family intramembrane metalloprotease. Science.

[15] Kanehara K., Akiyama Y., Ito K. (2001). Characterization of the yaeL gene product and its S2P-protease motifs in *Escherichia coli*. Gene.

[16] Alba B.M., Leeds J.A., Onufryk C., Lu C.Z., Gross C.A. (2002). DegS and YaeL participate sequentially in the cleavage of RseA to activate the ςE-dependent extracytoplasmic stress response. Genes Dev..

[17] Kanehara K., Ito K., Akiyama Y. (2002). YaeL (EcfE) activates the sigma(E) pathway of stress response through a site-2 cleavage of anti-sigma(E), RseA. Genes Dev..

[18] Alba B.M., Gross C.A. (2004). Regulation of the *Escherichia coli* sigma-dependent envelope stress response. Mol. Microbiol..

[19] Flynn J.M., Levchenko I., Sauer R.T., Baker T.A. (2004). Modulating substrate choice: the SspB adaptor delivers a regulator of the extracytoplasmic-stress response to the AAA+ protease ClpXP for degradation. Genes Dev..

[20] Akiyama K., Mizuno S., Hizukuri Y., Mori H., Nogi T., Akiyama Y. (2015). Roles of the membrane-reentrant β-hairpin-like loop of RseP protease in selective substrate cleavage. Elife.

[21] Hizukuri Y., Oda T., Tabata S., Tamura-Kawakami K., Oi R., Sato M. (2014). A structure-based model of substrate discrimination by a noncanonical PDZ tandem in the intramembrane-cleaving protease RseP. Structure.

[22] Akiyama K., Hizukuri Y., Akiyama Y. (2017). Involvement of a conserved GFG motif region in substrate binding by RseP, an *Escherichia coli* S2P protease. Mol. Microbiol..

[23] Parrell D., Zhang Y., Olenic S., Kroos L. (2017). *Bacillus subtilis* intramembrane protease RasP activity in *Escherichia coli* and *in vitro*. J. Bacteriol..

[24] Koide K., Ito K., Akiyama Y. (2008). Substrate recognition and binding by RseP, an *Escherichia coli* intramembrane protease. J. Biol. Chem..

[25] Reinseth I., Tønnesen H.H., Carlsen H., Diep D.B. (2021). Exploring the therapeutic potenital of the leaderless enterocins K1 and EJ97 in the treatment of vancomycin-resistant enterococcal infection. Front. Microbiol..

[26] Ovchinnikov K.V., Kristiansen P.E., Uzelac G., Topisirovic L., Kojic M., Nissen-Meyer J. (2014). Defining the structure and receptor binding domain of the leaderless bacteriocin LsbB. J. Biol. Chem..

[27] Varahan S., Iyer V.S., Moore W.T., Hancock L.E. (2013). Eep confers lysozyme resistance to Enterococcus faecalis via the activation of the extracytoplasmic function sigma factor SigV. J. Biol. Chem..

[28] An F.Y., Sulavik M.C., Clewell D.B. (1999). Identification and characterization of a determinant (eep) on the *Enterococcus faecalis* chromosome that is involved in production of the peptide sex pheromone cAD1. J. Bacteriol..

[29] Frank K.L., Guiton P.S., Barnes A.M., Manias D.A., Chuang-Smith O.N., Kohler P.L. (2013). AhrC and Eep are biofilm infection-associated virulence factors in *Enterococcus faecalis*. Infect. Immun..

[30] Akiyama Y., Kanehara K., Ito K. (2004). RseP (YaeL), an *Escherichia coli* RIP protease, cleaves transmembrane sequences. EMBO J..

[31] Sørvig E., Grönqvist S., Naterstad K., Mathiesen G., Eijsink V.G.H., Axelsson L. (2003). Construction of vectors for inducible gene expression in *Lactobacillus sakei* and *L. plantarum*. FEMS Microbiol. Lett..

[32] Sørvig E., Mathiesen G., Naterstad K., Eijsink V.G.H., Axelsson L. (2005). High-level, inducible gene expression in *Lactobacillus sakei* and *Lactobacillus plantarum* using versatile expression vectors. Microbiology (Reading).

[33] Kranjec C., Kristensen S.S., Bartkiewicz K.T., Brønner M., Cavanagh J.P., Srikantam A. (2021). A bacteriocin-based treatment option for *Staphylococcus haemolyticus* biofilms. Sci. Rep..

[34] Koide K., Maegawa S., Ito K., Akiyama Y. (2007). Environment of the active site region of RseP, an *Escherichia coli* regulated intramembrane proteolysis protease, assessed by site-directed cysteine alkylation. J. Biol. Chem..

[35] Miljkovic M., Uzelac G., Mirkovic N., Devescovi G., Diep D.B., Venturi V. (2016). LsbB bacteriocin interacts with the third transmembrane domain of the YvjB receptor. Appl. Environ. Microbiol..

[36] Dartigalongue C., Loferer H., Raina S. (2001). EcfE, a new essential inner membrane protease: its role in the regulation of heat shock response in *Escherichia coli*. EMBO J..

[37] Zhang Y., Luethy P.M., Zhou R., Kroos L. (2013). Residues in conserved loops of intramembrane metalloprotease SpoIVFB interact with residues near the cleavage site in pro-σK. J. Bacteriol..

[38] Olenic S., Buchanan F., VanPortfliet J., Parrell D., Kroos L. (2022). Conserved proline residues of *Bacillus subtilis* intramembrane metalloprotease SpoIVFB are important for substrate interaction and cleavage. J. Bacteriol..

[39] Callaway E. (2020). 'It will change everything': DeepMind's AI makes gigantic leap in solving protein structures. Nature.

[40] Urban S. (2009). Making the cut: central roles of intramembrane proteolysis in pathogenic microorganisms. Nat. Rev. Microbiol..

[41] Fredriksen L., Kleiveland C.R., Hult L.T.O., Lea T., Nygaard C.S., Eijsink V.G.H. (2012). Surface display of N-terminally anchored invasin by *Lactobacillus plantarum* activates NF-κB in monocytes. Appl. Environ. Microbiol..

[42] Aukrust T., Blom H. (1992). Transformation of Lactobacillus strains used in meat and vegetable fermentations. Food Res. Int..

[43] Dobson L., Reményi I., Tusnády G.E. (2015). Cctop: a consensus constrained TOPology prediction web server. Nucleic Acids Res..

[44] Mistry J., Chuguransky S., Williams L., Qureshi M., Salazar G.A., Sonnhammer E.L. (2021). Pfam: the protein families database in 2021. Nucleic Acids Res..

[45] Holo H., Nilssen Ø., Nes I. (1991). Lactococcin A, a new bacteriocin from *Lactococcus lactis* subsp. cremoris: isolation and characterization of the protein and its gene. J. Bacteriol..

[46] Jumper J., Evans R., Pritzel A., Green T., Figurnov M., Ronneberger O. (2021). Highly accurate protein structure prediction with AlphaFold. Nature.

[47] Salentin S., Schreiber S., Haupt V.J., Adasme M.F., Schroeder M. (2015). Plip: fully automated protein–ligand interaction profiler. Nucleic Acids Res..

[48] Wang J., Youkharibache P., Zhang D., Lanczycki C.J., Geer R.C., Madej T. (2020). iCn3D, a web-based 3D viewer for sharing 1D/2D/3D representations of biomolecular structures. Bioinformatics.

[49] Wang J., Youkharibache P., Marchler-Bauer A., Lanczycki C., Zhang D., Lu S. (2022). iCn3D: from web-based 3D viewer to structural analysis tool in batch mode. Front Mol. Biosci..

[50] Wiull K., Boysen P., Kuczkowska K., Moen L.F., Carlsen H., Eijsink V.G.H. (2022). Comparison of the immunogenic properties of Lactiplantibacillus plantarum carrying the mycobacterial Ag85B-ESAT-6 antigen at various cellular localizations. Front. Microbiol..

[51] Rice P., Longden I., Bleasby A. (2000). Emboss: the European molecular biology open software suite. Trends Genet..

[52] Robert X., Gouet P. (2014). Deciphering key features in protein structures with the new ENDscript server. Nucleic Acids Res..

[53] Kleerebezem M., Boekhorst J., van Kranenburg R., Molenaar D., Kuipers O.P., Leer R. (2003). Complete genome sequence of *Lactobacillus plantarum* WCFS1. Proc. Natl. Acad. Sci. U. S. A..

[54] Herranz C., Casaus P., Mukhopadhyay S., Martınez J., Rodrıguez J., Nes I. (2001). *Enterococcus faecium* P21: a strain occurring naturally in dry-fermented sausages producing the class II bacteriocins enterocin A and enterocin B. Food Microbiol..

