# Supplementary material for: The extracellular domain of site-2-metalloprotease RseP is important for sensitivity to bacteriocin EntK1
Source: J Biol Chem. 2022 Oct 14;298(11):102593. doi: 10.1016/j.jbc.2022.102593 (PMC9672952; doi:10.1016/j.jbc.2022.102593)
Supplement: Supplemental Tables [file mmc1.docx]

**Table S1| Primers used in this study.** Amino acid substitutions are highlighted in bold and underlined.

| **Name** | **Sequence (5’-3’)** |
| --- | --- |
| In-Fusion primers for amplification of *rseP* | |
| EfmRseP_F | GAGTATGATTCATATGAAAACGATTCTGACATTTATC |
| EfmRseP_R | TCGAACCCGGGGTACCCTAGAAAAAGAATCGTTGAATATCGTTCC |
| EfmRseP6His_R | CGAACCCGGGGTACCCTAATGATGATGATGATGATGGAAAAAGAATCGTTG |
| LlsRseP_RseP_F | GGAGTATGATTCATATGATAGAAACACTGATTACTTTTATT |
| LlsRseP_RseP_R | TCGAACCCGGGGTACCTTAATTTACAAAGGCTCGGAGAATATC |
| EfsRseP_F | GGAGTATGATTCATATGAAAACAATTATCACATTCATTATT |
| EfsRseP_R | TCGAACCCGGGGTACCTTAAAAGAAAAAGCGTTGAATATCGTTC |
| LpRseP_F | GGAGTATGATTCATATGATCGTTACAATTATTACGTTCATTA |
| LpRseP_R | CTGTAATTTGAAGCTTTTAGAAGAAATATCGCTGAATATCATTC |
| LpRseP6His_R | CTGTAATTTGAAGCTTTTAATGATGATGATGATGATGGAAGAAATATCGCTGAATATCATTC |
| SasRseP_F | GGAGTATGATTCATATGGTGAGCTATTTAGTTACAATAATTGCAT |
| SasRseP_R | TCGAACCCGGGGTACCTTATAAGAAATATCGTCGAATATCATTC |
| SaeRseP_F | GGAGTATGATTCATATGATAAAAATACGAGGTGTAGTTAATTTGA |
| SaeRseP_R | TCGAACCCGGGGTACCTTATAAGAAATAACGTTGTATATCATTCCTTCC |
| Primers for site-directed mutagenesis | |
| EfmH18A_F | TCTGACATTTATCATCGTTTTTGGTATATTAGTGATTGTT**GCG**GAGTTTGGTCATTTCTTCTTT |
| EfmH18A_R | AAAGAAGAAATGACCAAACTC**CGC**AACAATCACTAATATACCAAAAACGATGATAAATGTCAGA |
| EfmE19A_F | GGTATATTAGTGATTGTTCAT**GCG**TTTGGTCATTTCTTCTTTGCG |
| EfmE19A_R | CGCAAAGAAGAAATGACCAAA**CGC**ATGAACAATCACTAATATACC |
| EfmH22A_F | ATATTAGTGATTGTTCATGAGTTTGGT**GCG**TTCTTCTTTGCGAAACGATCAGGAATC |
| EfmH22A_R | GATTCCTGATCGTTTCGCAAAGAAGAA**CGC**ACCAAACTCATGAACAATCACTAATAT |
| EfmAAxxA_F | CGATTCTGACATTTATCATCGTTTTTGGTATATTAGTGATTGTT**GCGGCG**TTTGGT**GCG**TTCTTCTTTGCGAAACGATCAGG |
| EfmAAxxA_R | CCTGATCGTTTCGCAAAGAAGAA**CGC**ACCAAA**CGCCGC**AACAATCACTAATATACCAAAAACGATGATAAATGTCAGAATCG |
| EfmN359A_F | GATGGCGCTTCTTTCAATG**GCT**CTCGGAATCGTCAATCTG |
| EfmN359A_R | CAGATTGACGATTCCGAG**AGC**CATTGAAAGAAGCGCCATC |
| EfmN364A_F | AATGAATCTCGGAATCGTC**GCT**CTGCTTCCGATTCCTGCC |
| EfmN364A_R | GGCAGGAATCGGAAGCAG**AGC**GACGATTCCGAGATTCATT |
| EfmP367A_F | CGGAATCGTCAATCTGCTT**GCG**ATTCCTGCCTT |
| EfmP367A_R | AAGGCAGGAAT**CGC**AAGCAGATTGACGATTCCG |
| EfmP369A_F | CGTCAATCTGCTTCCGATT**GCT**GCCTTAGATGG |
| EfmP369A_R | CCATCTAAGGC**AGC**AATCGGAAGCAGATTGACG |
| Efm_D372A_F | TGCTTCCGATTCCTGCCTTA**GCT**GGCGGGAAATTA |
| Efm_D372A_R | TAATTTCCCGCC**AGC**TAAGGCAGGAATCGGAAGCA |
| Primers used for construction of *rseP* hybrids | |
| Hyb1_F | GCCCGTACGGCCGGTTTAAAAGAAAATGATGAGGTAGTCAGTGT |
| Hyb1_R | ATCATTTTCTTTTAAACCGGCCGTACGGG |
| Hyb2_F | CACGGGTTCAGTTTGGATAAATTAGGCGGACCTGTCA |
| Hyb2_R | TCCGCCTAATTTATCCAAACTGAACCCGTGAGTGA |
| Hyb3_F | TGCGGCAGAAGCAGGCATTCAAAAGGGCGATCAAATC |
| Hyb3_R | TCGCCCTTTTGAATGCCTGCTTCTGCCGCA |
| LpRseP_XmaI_R | TTGGCGCCTTCGAACCCGGGTTAATGATGATGATGATGATGGAAGAAATATCGCT |
| Hyb4_F | ACAGGTTTCAGTTTAAACGATTTAGGTGGGCC |
| Hyb4_R | CCCACCTAAATCGTTTAAACTGAAACCTGTAAATAGTGATC |
| Hyb5_F | ATGCAAGGTGGTGTTACGAGTACAACGACCCA |
| Hyb5_R | GGTCGTTGTACTCGTAACACCACCTTGCATAAATGCCA |
| Hyb6_F | TCGGCTAAATTGTGGCAACGAATGTTGACGAATTTTGC |
| Hyb6_R | CGTCAACATTCGTTGCCACAATTTAGCCGATTGGA |
| Hyb7_F | CGATCAGGAATCCTCGTGCGTGAATTTTCTGTCGGGA |
| Hyb7_F2 | CGATCAGGAATCCTCGTGCGTGAATTTTCTGTCGGGA |
| Hyb8_R | GTTGGTCAGCATACGTTGCCATAACTTGGCCGATT |
| Hyb8_F | GCCAAGTTATGGCAACGTATGCTGACCAACTTTGC |
| Hyb9a_R | AGTGTTCGTCACTTGAACACCACCCTGCATAAA |
| Hyb9b_F | ATGCAGGGTGGTGTTCAAGTGACGAACACTAATCGC |
| Primers used for construction of truncated *rseP* | |
| Trunc_F | GCGAATTTGCTATCAAAGACGTACAGTTCCAATCGGCT |
| Trunc_R | GGAACTGTACGTCTTTGATAGCAAATTCGCGGACGA |

**Table S2| Amino acids sequences employed in AlphaFold-Multimer and structure analysis**

| **Name** | **Sequence** |
| --- | --- |
|  |  |
| EntK1 | MKFKFNPTGTIVKKLTQYEIAWFKNKHGYYPWEIPRC |
| RseP from *E. faecium* | MKTILTFIIVFGILVIVHEFGHFFFAKRSGILVREFAIGMGPKIYGHQAKDGTTYTLRLLPIGGYVRMAGNGDDETEMAPGMPLSLLLNSDGIVEKINLSKKIQLTNAIPMELSRYDLEDELTITGYVNGDETEVVTYPVDHDATIIENDGTEIRIAPKDVQFQSAKLWQRMLTNFAGPMNNFILAIVLFIILAFMQGGVQVTNTNRVGEIMPNGAAAEAGLKENDEVVSVDGKEIHSWNDLTTVITKNPGKTLDFKIEREGQVQSVDVTPKSVESNGEKVGQLGIKAPMNTGFMDKIIGGTRQAFSGSLEIFKALGSLFTGFSLDKLGGPVMMYQLSSEAANQGITTVISLMALLSMNLGIVNLLPIPALDGGKLVLNIFEGIRGKPLSQEKEGILTLAGFGFLMLLMVLVTWNDIQRFFF |
