# Supplementary material for: The extracellular domain of site-2-metalloprotease RseP is important for sensitivity to bacteriocin EntK1
Source: J Biol Chem. 2022 Oct 14;298(11):102593. doi: 10.1016/j.jbc.2022.102593 (PMC9672952; doi:10.1016/j.jbc.2022.102593)
Supplement: Figure S1 — Pairwise sequence alignment of EfmRseP and LpRseP. Identical amino acids are shaded in black while amino acids of similar chemical properties are boxed. Alignment was generated using EMBOSS Needle and the figure was exported using ESPript 3 web-server (51, 52). [file mmc2.pdf]

```
            1        10        20        30        40        50
1 EfmRseP   .MKTILTFIIVFGILVIVHEFGHFFFAKRSGILVREFAIGMGPKIYGHQAKDGT
2 LpRseP    MIVTIITFIIVFGILVIVHEFGHFYFAKKAGILVREFSVGMGPKAVAFR.RNAT

                60        70        80        90        100
1 EfmRseP   TYTLRLLPIGGYVRMAGNGDDE.TEMAPGMPLSLLLNSDGIVEKINLSKKIQLT
2 LpRseP    TYTLRFLPIGGYVRMAGVADDEDEELKPGTPVSLQIGPDGIVHSINASKKTTLF

             110       120       130       140       150       160
1 EfmRseP   NAIPMELSRYDLEDELTITGYVNGDETEVVTYPVDHDATIIENDGTEIRIAPKD
2 LpRseP    NGIPLSVTATDLEKELWIEGYENGDESEVKHYAVDHDATIVESDGTEVQIAPVD

                 170       180       190       200       210
1 EfmRseP   VQFQSAKLWQRMLTNFAGPMNNFILAIVLFIILAFMQGGVQVTNTNRVGEIMPN
2 LpRseP    VQFQSAKLWQRMLTNFAGPMNNFILAIITFAILAFMQGGVTSTTTH.VAATTAD

                220       230       240       250       260
1 EfmRseP   GAAAEAGLKENDEVVSVDGKEIHSWNDLTTVITKNPGKTLDFKIEREGQVQSVD
2 LpRseP    SVARTAGIQKGDQIVAVNGKKMTSAQSISLLIQDSPKQRLTLTINRAGQTKKIA

             270       280       290       300            310
1 EfmRseP   VTPKSVESNGEKVGQLGIKAPMNTGFMDKIIGG.TRQAFSGS....LEIFKALG
2 LpRseP    VTPAAKTVSGNRIGQIGVQWATKT...DTSLGAKLAYGFTGSWGITKQIFQVLG

               320       330       340       350       360       370
1 EfmRseP   SLFT.GFSLDKLGGPVMMYQLSSEAANQGVTTVISLMALLSMNLGIVNLLPIPA
2 LpRseP    RMVTHGFSLNDLGGPVAIFATTSQAAKSGVRTVIYLLAVLSINLGIVNLLPIPA

                 380       390       400       410       420
1 EfmRseP   LDGGKLVLNIFEGIRGKPLSQEKEGILTLAGFGFLMLLMVLVTWNDIQRFFF
2 LpRseP    LDGGKLLLNIVEGIRGKPLRVETESVITLIGFGLLMLLMILVTWNDIQRYFF
```
