# Supplementary figures and images for: The extracellular domain of site-2-metalloprotease RseP is important for sensitivity to bacteriocin EntK1
Source: J Biol Chem. 2022 Oct 14;298(11):102593. doi: 10.1016/j.jbc.2022.102593 (PMC9672952; doi:10.1016/j.jbc.2022.102593)

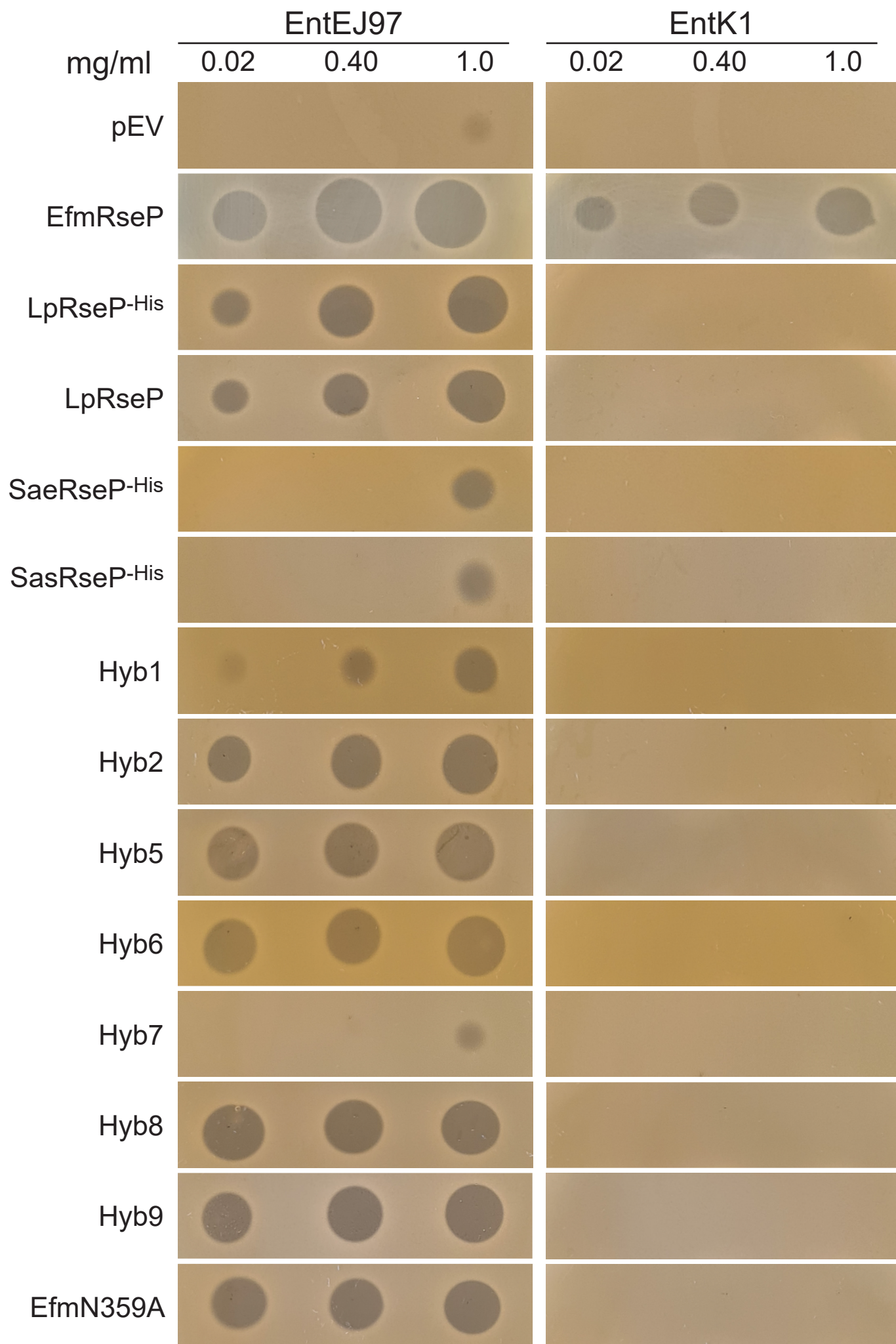

Supplement: Figure S2 — EntEJ97 inhibition zones in spot-on-lawn assays. 3 μl of EntEJ97 and EntK1 (4–220 μM) was spotted on lawns of L. plantarum clones expressing different modified versions of RseP. Since RseP is the receptor for EntEJ97, increased sensitivity towards EntEJ97 compared to the empty vector pEV may indicate proper production and folding of the target protein expression. An inhibition zone of >10 mm was observed for all hybrids and point mutants tested, except for Hyb7, SaeRseP, SasRseP and pEV. A zone comparable to pEV was observed for Hyb7, SaeRseP and SasRseP (<6 mm). [file mmc3.pdf]

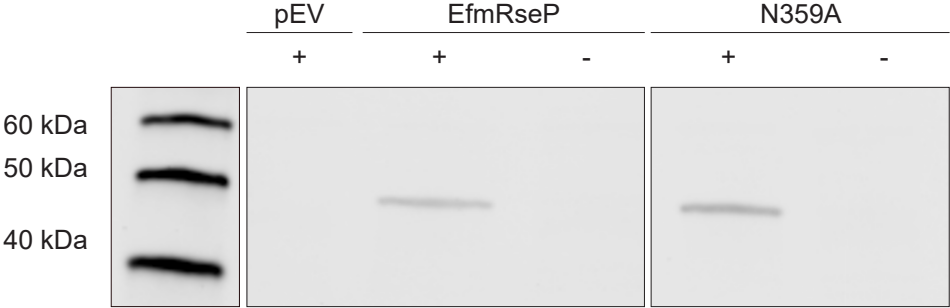

Supplement: Figure S4 — Western blot. pEV, EfmRseP and the N359A mutant under inducing (+) and non-inducing (-) conditions. Cells were harvested, lysed and subjected to SDS-PAGE as previously described (50), with minor modifications. The harvested cells were resuspended in 250 μl NP-40 lysis buffer containing 1 mM PMSF (phenylmethylsulfonyl fluoride). Samples where not boiled prior to SDS-PAGE. Following electrophoresis, proteins were electroblotted onto nitrocellulose mini membranes using iBlotTM Transfer Stack (Invitrogen) and the iBLot Gel transfer device (Invitrogen). The membrane was washed with Tris-Buffered Saline (TBS; 2 × 10 min), then subsequently incubated with blocking buffer (5% Bovine Serum Albumin (BSA) dissolved in TBS) for 1 h and washed again (2 × 10 min Tween-TBS (TTBS; 0.05% Tween-20), 1 × 10 min TBS). The membrane was incubated with Penta-His (Qiagen) (1:1000 in Blocking buffer) for 30 min, following an overnight incubation at 4 °C. The subsequent day the membrane was incubated for 30 min at room temperature, washed (2 × 10 min, TTBS), and incubated with the polyclonal HRP-conjugated anti-mouse IgG (Sigma-Aldrich) secondary antibody (1:5000) in blocking buffer for 1 h. To remove unbound secondary antibodies the membrane was washed 4 × 10 min with TTBS. The blots were subsequently visualized using the SuperSignal West Pico PLUS Chemiluminescent substrate (Thermo Fisher Scientific) and image using the Azure c400 system (Azure Biosystem, Dublin, CA). [file mmc6.pdf]
