# Supplementary material for: The extracellular domain of site-2-metalloprotease RseP is important for sensitivity to bacteriocin EntK1
Source: J Biol Chem. 2022 Oct 14;298(11):102593. doi: 10.1016/j.jbc.2022.102593 (PMC9672952; doi:10.1016/j.jbc.2022.102593)
Supplement: Figure S3A — Schematic representation of all RseP hybrids and truncated RseP. Hybrid proteins in which parts of LpRseP (from the EntK1-insensitive L. plantarum, indicated in purple) were replaced with the corresponding sequence from EfmRseP (from the EntK1-sensitive E. faecium, indicated in green), were constructed. Conserved S2P motifs are indicated (HExxH, GxG, MRE-ß, PDZ, LDG). TMS1-4 indicate the four predicted transmembrane segments (TMS), while the black arrows indicate the point(s) of fusion between LpRseP and EfmRseP regions. The gray dashed line indicates part of the full-length EfmRseP deleted in Trunc. Protein variants giving a MIC50 ≥22 μM EntK1 are marked as fully EntK1-resistant, while protein variants giving MIC50 values below 2.7 μM are marked as EntK1-sensitive. Note that sensitivity data for Hyb7 are uncertain due to possible expression issues visible in the control experiment of Fig. S2. For MIC50 values, see Table 3. [file mmc4.pdf]

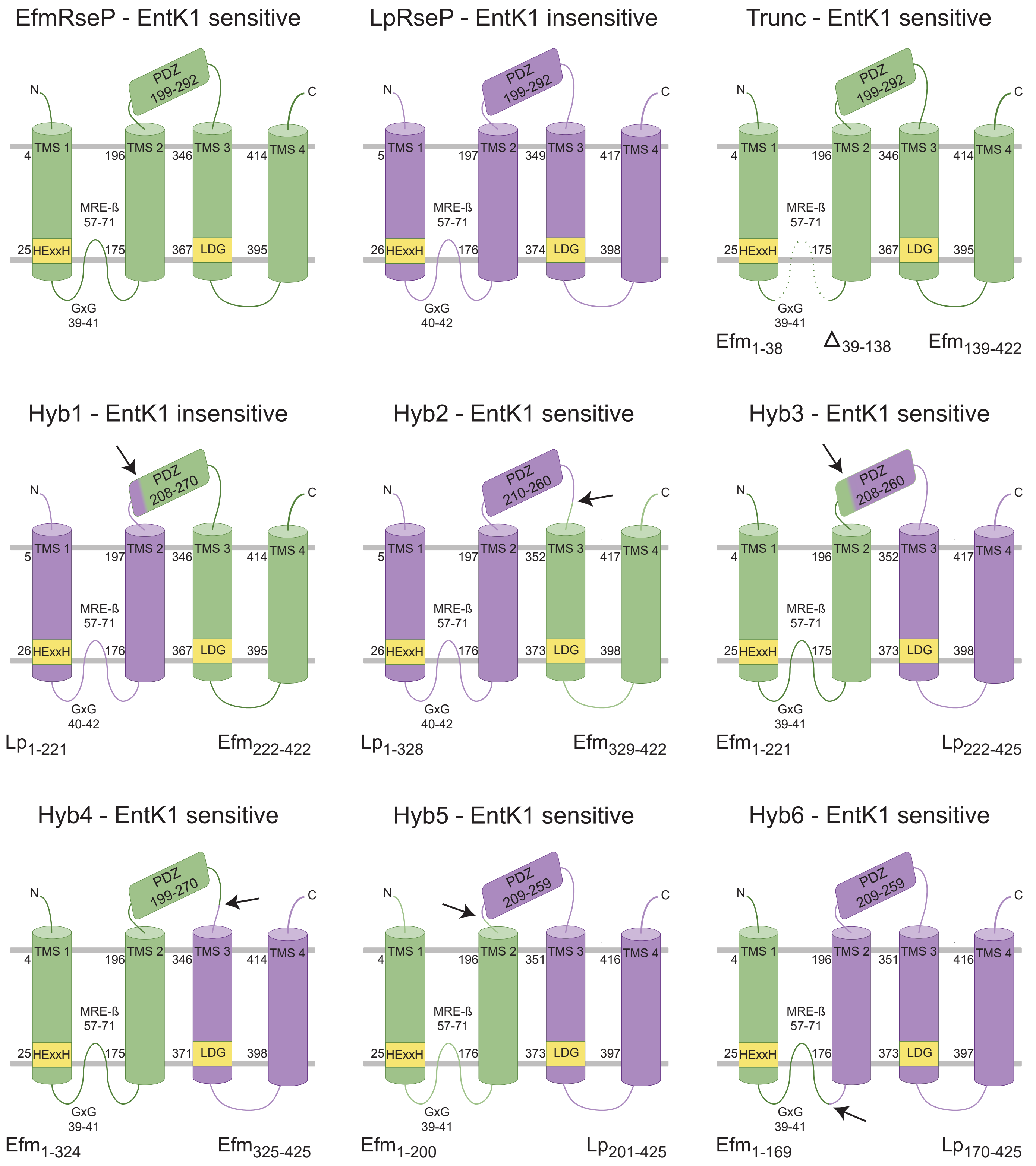

EfmRseP - EntK1 sensitive
N
C
PDZ 199-292
TMS 1
TMS 2
TMS 3
TMS 4
4
196
346
414
MRE-ß 57-71
25 HExxH
175
367 LDG
395
GxG 39-41
LpRseP - EntK1 insensitive
N
C
PDZ 199-292
TMS 1
TMS 2
TMS 3
TMS 4
5
197
349
417
MRE-ß 57-71
26 HExxH
176
374 LDG
398
GxG 40-42
Trunc - EntK1 sensitive
N
C
PDZ 199-292
TMS 1
TMS 2
TMS 3
TMS 4
4
196
346
414
MRE-ß 57-71
25 HExxH
175
367 LDG
395
GxG 39-41
Efm_{1-38}
Δ_{39-138}
$Efm_{139-422}$
Hyb1 - EntK1 insensitive
N
C
PDZ 208-270
TMS 1
TMS 2
TMS 3
TMS 4
5
197
346
414
MRE-ß 57-71
26 HExxH
176
367 LDG
395
GxG 40-42
Lp_{1-221}
$Efm_{222-422}$
Hyb2 - EntK1 sensitive
N
C
PDZ 210-260
TMS 1
TMS 2
TMS 3
TMS 4
5
197
352
417
MRE-ß 57-71
26 HExxH
176
373 LDG
398
GxG 40-42
Lp_{1-328}
$Efm_{329-422}$
Hyb3 - EntK1 sensitive
N
C
PDZ 208-260
TMS 1
TMS 2
TMS 3
TMS 4
4
196
352
417
MRE-ß 57-71
25 HExxH
175
373 LDG
398
GxG 39-41
Efm_{1-221}
$Lp_{222-425}$
Hyb4 - EntK1 sensitive
N
C
PDZ 199-270
TMS 1
TMS 2
TMS 3
TMS 4
4
196
346
414
MRE-ß 57-71
25 HExxH
175
371 LDG
398
GxG 39-41
Efm_{1-324}
$Efm_{325-425}$
Hyb5 - EntK1 sensitive
N
C
PDZ 209-259
TMS 1
TMS 2
TMS 3
TMS 4
4
196
351
416
MRE-ß 57-71
25 HExxH
176
373 LDG
397
GxG 39-41
Efm_{1-200}
$Lp_{201-425}$
Hyb6 - EntK1 sensitive
N
C
PDZ 209-259
TMS 1
TMS 2
TMS 3
TMS 4
4
196
351
416
MRE-ß 57-71
25 HExxH
176
373 LDG
397
GxG 39-41
Efm_{1-169}
$Lp_{170-425}$
