# Supplementary material for: The extracellular domain of site-2-metalloprotease RseP is important for sensitivity to bacteriocin EntK1
Source: J Biol Chem. 2022 Oct 14;298(11):102593. doi: 10.1016/j.jbc.2022.102593 (PMC9672952; doi:10.1016/j.jbc.2022.102593)
Supplement: Figure S3B [file mmc5.pdf]

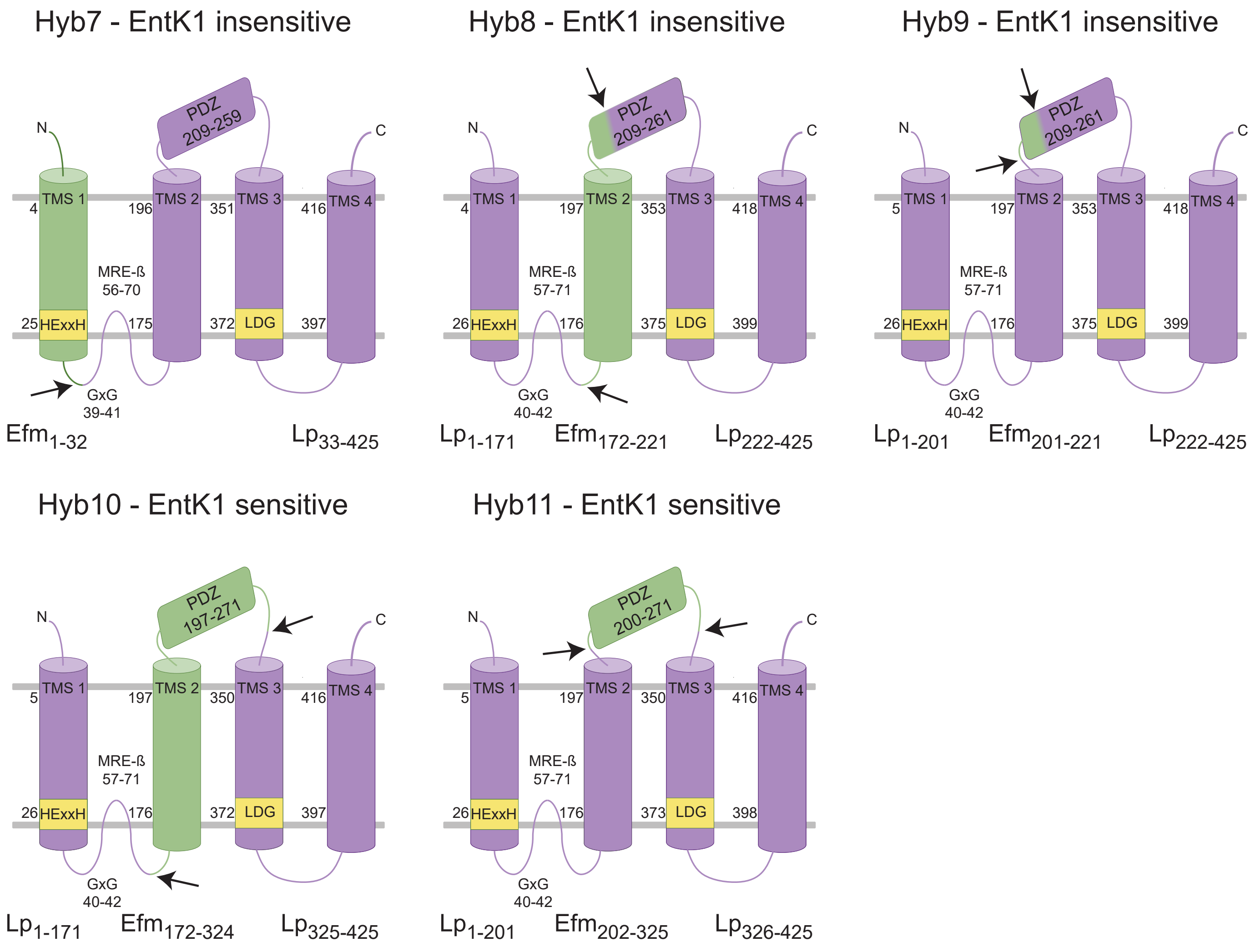
Hyb7 - EntK1 insensitive
N
C
PDZ
209-259
TMS 1
TMS 2
TMS 3
TMS 4
4
196
351
416
MRE-ß
56-70
25
HExxH
175
372
LDG
397
GxG
39-41
Efm1-32
Lp33-425
Hyb8 - EntK1 insensitive
N
C
PDZ
209-261
TMS 1
TMS 2
TMS 3
TMS 4
4
197
353
418
MRE-ß
57-71
26
HExxH
176
375
LDG
399
GxG
40-42
Lp1-171
Efm172-221
Lp222-425
Hyb9 - EntK1 insensitive
N
C
PDZ
209-261
TMS 1
TMS 2
TMS 3
TMS 4
5
197
353
418
MRE-ß
57-71
26
HExxH
176
375
LDG
399
GxG
40-42
Lp1-201
Efm201-221
Lp222-425
Hyb10 - EntK1 sensitive
N
C
PDZ
197-271
TMS 1
TMS 2
TMS 3
TMS 4
5
197
350
416
MRE-ß
57-71
26
HExxH
176
372
LDG
397
GxG
40-42
Lp1-171
Efm172-324
Lp325-425
Hyb11 - EntK1 sensitive
N
C
PDZ
200-271
TMS 1
TMS 2
TMS 3
TMS 4
5
197
350
416
MRE-ß
57-71
26
HExxH
176
373
LDG
398
GxG
40-42
Lp1-201
Efm202-325
Lp326-425
